# Transcriptional Activation of *Mina* by Sp1/3 Factors

**DOI:** 10.1371/journal.pone.0080638

**Published:** 2013-12-04

**Authors:** Shangli Lian, Hari Hara S. K. Potula, Meenu R. Pillai, Melanie Van Stry, Madoka Koyanagi, Linda Chung, Makiko Watanabe, Mark Bix

**Affiliations:** Department of Immunology, St. Jude Children’s Research Hospital, Memphis, Tennessee, United States of America; Peking University Health Science Center, China

## Abstract

Mina is an epigenetic gene regulatory protein known to function in multiple physiological and pathological contexts, including pulmonary inflammation, cell proliferation, cancer and immunity. We showed previously that the level of *Mina* gene expression is subject to natural genetic variation linked to 21 SNPs occurring in the *Mina* 5′ region [1]. In order to explore the mechanisms regulating *Mina* gene expression, we set out to molecularly characterize the *Mina* promoter in the region encompassing these SNPs. We used three kinds of assays – reporter, gel shift and chromatin immunoprecipitation – to analyze a 2 kb genomic fragment spanning the upstream and intron 1 regions flanking exon 1. Here we discovered a pair of *Mina* promoters (P1 and P2) and a P1-specific enhancer element (E1). Pharmacologic inhibition and siRNA knockdown experiments suggested that Sp1/3 transcription factors trigger Mina expression through additive activity targeted to a cluster of four Sp1/3 binding sites forming the P1 promoter. These results set the stage for comprehensive analysis of *Mina* gene regulation from the context of tissue specificity, the impact of inherited genetic variation and the nature of upstream signaling pathways.

## Introduction

The JmjC family protein Mina has been implicated in immune function, cell proliferation and cancer. Tsuneoka et al first discovered Mina as a 53 Kd Myc-induced nuclear antigen with the capacity to regulate cell proliferation [2], [3]. High level MINA expression in tumor biopsies has been linked to poor prognosis in a variety of human cancers. These include colon cancer, esophageal squamous cell carcinoma, gingival squamous cell carcinoma, renal cell carcinoma, lymphoma, neuroblastoma, gastric carcinoma, hepatocellular carcinoma and lung cancer [2], [4]–[13]. More recently, *Mina* was found to control T helper (Th) 2, Th17 and T regulatory cell differentiation [1], [14].

Given clear evidence of Mina’s involvement in immunity, cell proliferation and cancer, it is important to understand how Mina expression is regulated. We know from analysis of protein turnover and pre-mRNA transcription rate that Mina protein abundance is controlled largely at the transcriptional level [1]. However, the mechanisms governing *Mina* transcription remain poorly understood. To begin addressing this gap, we report here the molecular characterization of the *Mina* promoter region and its trans-acting factors in murine T cells. Using a dual luciferase reporter assay to interrogate nested deletions of a region spanning the *Mina* transcriptional start site (TSS), we defined a 144 bp minimal *Mina* promoter encompassing four potential Sp1/3 binding sites. Gel shift assays validated all sites as functional for Sp1 and Sp3 binding.

Furthermore, mutagenesis analysis demonstrated that full reporter activity required WT sequence at all 4 Sp1/3 binding sites. Pharmacological inhibition and siRNA knockdown of Sp1/3 binding activity and level, respectively, substantially diminished *Mina* mRNA expression. Finally, chromatin immunoprecipitation (ChIP) assays in primary T helper cells revealed the *Mina* promoter region to be enriched in bound Sp1 and Sp3 as well as lysine-4 trimethylated histone H3 (H3K4me3), a marker of transcriptionally active chromatin. Together, these results indicate a physiological requirement of Sp1 and Sp3 for *Mina* transcription and provide a stimulus for analysis of potential distal regulatory elements and the upstream pathways responsible for the tight regulation of Mina expression in its diverse physiological contexts.

## Materials and Methods

### Ethics Statement

Mice used in this study were maintained in specific pathogen-free conditions in accordance with the guidelines of the Institutional Animal Care and Use Committee of St. Jude Children’s Research Hospital under protocol 453 approved by the St. Jude Institutional Animal Care and Use Committee.

### Mice

BALB/c and C57BL/6 mice were purchased from Jackson Lab.

### Reagents and Antibodies

Anti-TCRβ was purified from hybridoma H57.597. Anti-CD28 was purchased from Biolegend (102102). Anti-Mina antibody was purchased from Zymed (clone M532). Anti-H3K4me3 (07–030) and anti-H3K27me3 (07–449) antibodies were purchased from Upstate (Millipore). Isotype control Rabbit IgG (AB46540-1), Mouse IgG (AB18413) and Goat IgG (AB37373) were purchased from Abcam. Sp1 (PEP 2, sc-59), Sp3 (D-20, sc-644), RUNX3 (sc-23576X), and YY1 (sc-1703X) were purchased from Santa Cruz. Mouse recombinant IL-2 (354078 BD) was used at 20 U/ml. Mithramycin A (M6891) was purchased from Sigma-Aldrich. Poly dA:dT (Cat# tlrl-patn) was purchased from InvivoGen. ChIP-grade Protein G Magnetic Beads (Cat#9006) were purchased from Cell Signaling.

### Cloning

A 2 kb Mina proximal promoter region (−1588 to +351) was PCR amplified from Mus musculus BAC clone RP23-23O4 (AC154854) using forward primer 5′-TCAATGAGAAAGGGGCCT-3′ and reverse primer 5′-CAACCTACGCTCCAAGTC-3′. The 2 kb fragment was then cloned into PGL3 basic vector (Promega) to drive firefly luciferase (FL) expression. 5′ and 3′ nested deletions were generated using the Erase-a-Base system (Promega). The Mina promoter fragment (−64∼+80) was amplified using forward primer 5′-GTGGTCCGGGGGCGGA-3′ and reverse primer 5′-AGTTGACCCAGCTAAG-3′, and then blunt end cloned into PGL3 basic vector. The Mina promoter fragment (-64∼+151) was amplified using forward primer 5′-ATATATGATATCGTGGTCCGGGGGCGGA-3′ and reverse primer 5′-ATATATGATATCAGAGCTGCACTTCTCAGCCTGA-3′, and then cloned into the EcoRV site of PGL3 basic vector. Mutagenesis of Sp1/3 binding sites was performed on Mina promoter (−64∼+151) using QuickChange II-E Site-Directed Mutagenesis Kit (Cat# 200555, Agilent Technologies).

### Cell Culture

EL4 cells were cultured in RPMI containing 10% FBS, penicillin/streptomycin (GIBCO 15140), L-Glu (GIBCO 25030) and β-mercaptoethanol (GIBCO 21985). 1×10^6^ EL4 cells were treated for 24 h with 10 nM, 100 nM, 1 µΜ Mithramycin or 1 μΜ DMSO as carrier control.

### Luciferase Assay

Lipofectamine LTX with Plus reagent (Invitrogen) was used to cotransfect EL4 cells (2×10^5^) with PGL3 reporter constructs expressing firefly luciferase (FL) (750 ng) and pRL-TK expressing control renilla luciferase (RL) (40 ng). Following 48 h culture in 24 well plates, cells were harvested and assayed for FL and RL activity using the Dual-Luciferase Reporter Assay System (Promega).

### Electrophoretic Mobility Shift Assay

EL4 cell nuclear extracts were prepared with the NER-PER extraction kit (Pierce, 78833). Protein concentrations were determined using the Bradford Assay (Thermo Scientific, 1856209) and bovine serum albumin as a standard (Sigma A9418). Probes were generated by annealing 5′ biotin-labeled oligonucleotides at 95°C in annealing buffer (100 mM Tris pH 7.5, 10 mM EDTA, 2 M NaCl, 50 mM MgCl2) followed by slow cooling to 22°C (∼3 hrs). Probe sequences are given in Table S1. Nuclear extracts (10 µg), Poly (dA:dT) (1 µg), and biotinylated probes (200 fmol) were incubated together at 22°C for 30 min in 20 µl binding buffer (10 mM Tris pH7.5, 60 mM KCl, 2 mM MgCl2, 0.15 mM dithiothreitol). For competition, 100-fold molar excess of unlabeled probe (20 pmol) was added to the reaction before adding biotinylated probe. For supershift, antibody (2 µg) was included in the reaction for 15 min before biotinylated probe was added. Binding reactions were resolved on 0.5X TBE 4% polyacrylamide gels at 150 V for 2–3 hr, transferred to nitrocellulose for 30 min at 4°C, and then UV crosslinked for 45–60 second using the auto crosslink function of the UV-light crosslinking instrument (Stratagene). Probe signals were detected using the Chemiluminescent Nucleic Acid Detection Module kit (Cat# 89880, Pierce).

### Chromatin Immunoprecipitation (ChIP) Assay

EL4 cells were fixed in 1% formaldehyde for 15 min at RT. 10×10^6^ nuclei (isolated as described for FAIRE in [15]) were resuspended in 350 µl Buffer 3 (21) and sonicated using a BioRuptor (Diagenode) until the average chromatin fragment size was ∼500 bp. Sonicated samples were centrifuged at 16,000 g for 10 min at RT and supernatants diluted 1 to 8 in dilution buffer (1% Triton X-100, 2 mM EDTA, 150 mM NaCl, 20 mM TrisHCl pH8, 1X protease inhibitor). Immunoprecipitation was performed by combining 1 ml of diluted supernatant (∼4×10^6^ nuclei) with 4 µg of antibody or corresponding IgG control and overnight rotation at 4°C. Then 30 µl of Protein G magnetic beads was added and rotation continued for another 3 hours at 4°C. Beads were separated by incubation on a magnet rack and washed sequentially with 1 ml of the following buffers: Par Wash 1# (1% SDS, 1% Triton X-100, 2 mM EDTA, 20 mM TrisHCl pH8, 150 mM NaCl, 1X protease inhibitor), Par Wash 2# (1% SDS, 1% Triton X-100, 2 mM EDTA, 20 mM TrisHCl pH8, 500 mM NaCl, 1X protease inhibitor), Par Wash 3# (1 mM EDTA, 10 mM TrisHCl pH8, 250 mM LiCl, 1% NP-40, 1% DOC, 1X protease inhibitor), and TE buffer. Each wash was performed by 5 min rotation at RT. Elution of DNA/protein complexes was performed by adding 100 µl freshly made elution buffer (0.1 M NaHCO3/1% SDS), vortexing, and 10 min rotation at RT. A second elution was combined with the first for a total volume of 200 µl. Crosslinks were reversed by adding 8 µl of 5 M NaCl to the combined eluates followed by overnight incubation at 65°C. DNA was purified by adding 20 µl of protein digestion buffer (0.4 M Tris-HCl, pH 6.8, 0.1 M EDTA, 0.8 mg/ml Proteinase K), mixing, incubation for 1 h at 50°C and phenol/chloroform extraction. The purified DNA pellet was dried and resuspended in 20 µl of water. Quantitative real time PCR was performed to measure the relative enrichment of Mina promoter and Mina intron 2 DNA sequences. Primer sequences are given in Table S1.

For ChIP performed with CD4 T cells, lymphocytes were isolated from spleen and lymph nodes of C57BL/6 or BALB/c mice, sorted for CD4+/CD62L+/CD44−/CD25− T cells, and cultured for 6–8 h in 6-well plates coated with 0.1 µg/ml anti-TCRβ and 10 µg/ml anti-CD28 (Biolegend). Cells were harvested and fixed in 1% formaldehyde at RT for 10 min and then processed as described above.

### Quantitative Real-Time (RT) PCR

Total RNA was extracted with TRIzol reagent (Invitrogen) and cDNA synthesized using SuperScript III Reverse Transcriptase (Invitrogen). Quantitative real-time PCR was performed using iQ SYBR Green Supermix on Real-Time Detection System (Applied Biosytems) or MX4000 real-time PCR system (Stratagene). Primers for detecting Mina, Gapdh and Hprt were described before [1]. Primers for Sp1 and Sp3 are as follows: Sp1 Forward 5′-ACCATGAGCGACCAAGATCA-3′ and Sp1 Reverse 5′-CATTGCCGCTACCCCCATTA-3′; Sp3 Forward 5′-ACCCTGAAGAATGGCAGCTC-3′ and Sp3 Reverse 5′-GGTACCTCTCCCACCACCTT-3′. mRNA level was normalized to house keeping genes Hprt and Gapdh.

### siRNA Interference

EL4 cells were transfected in 96-well plates with Accell siRNA transfection reagent (Thermo scientific Dharmacon; non-specific siRNA, D-001910-10-05; Sp1 siRNA, E-040633-00-0005; Sp3 siRNA, E-040397-00-0005) and cultured for 72 hours prior to RNA harvest and analysis by quantitative RT-PCR.

## Results

### Two Promoters and an Enhancer in the Mina TSS-proximal Region

**Figure 1:**
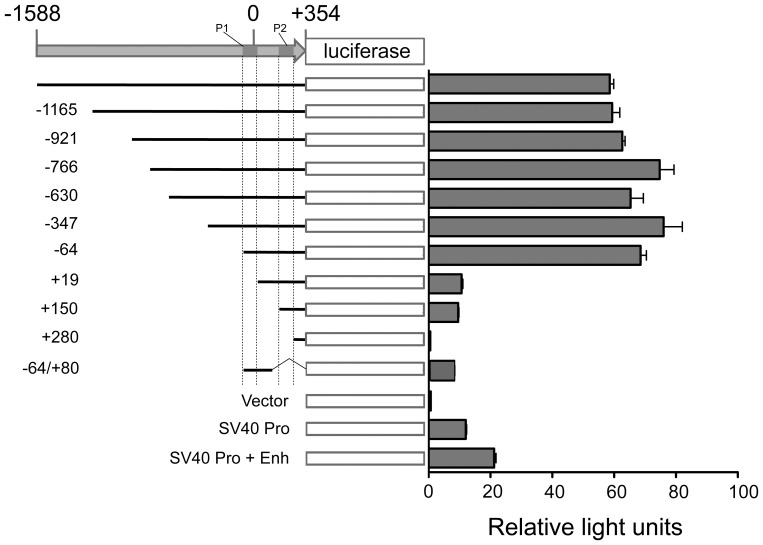
Reporter assay analysis of a 2 kb *Mina* promoter fragment. The transcriptional start site (TSS), labeled 0, is from NCBI (NM_025910.3). Internal control plasmid pRL-TK encoding renilla luciferase was cotransfected with each firefly luciferase construct. Parental (light gray filled arrow) and nested deletion constructs are diagrammed to the left of the bar graph with numbers representing the 5′ (and in the case of −64/+80, the 3′) termini of each construct. The inferred positions of P1 and P2 are labeled and depicted as dark gray rectangles. Vector is PGL3 basic vector; SV40 pro is PGL3 basic vector containing the SV40 promoter; and SV40 pro+enh is PGL3 basic vector containing the SV40 promoter and enhancer elements. Data are expressed as relative light units (the ratio of firefly to renilla luciferase activity) and presented as the mean and SEM of three independent experiments.

To map the *Mina* promoter, a ∼2 kb-fragment spanning the transcriptional start site (TSS) from position −1588 to +354 was cloned into PGL3 basic vector and tested for reporter activity by dual luciferase assay in the thymoma cell line EL4 (Fig. 1, gray filled arrow). Fragment (−1588/+354) contained strong reporter activity, respectively ∼6- and ∼60-fold higher than that driven by the SV40 promoter and by vector alone (Fig. 1). To characterize this activity, we interrogated a panel of 5′ nested deletions of fragment (−1588/+354) by reporter assay. Whereas deletions extending as far as −64 had no impact, removal of an additional 83 base pairs to position +19 caused a dramatic drop but not complete abolition of activity (Fig. 1). These results suggested: (1) that region (−64/+19) contains an element that mediates strong reporter activity suggestive of an enhancer; and (2) that fragment (+19/+354) contains a *Mina* promoter (termed P2; Fig. 1, rightmost dark gray rectangle) comparable in activity to the SV40 promoter. To further localize P2 within fragment (+19/+354), two additional 5′ deletions were generated and tested. One, extending as far as position +150, maintained P2 activity, whereas a second deletion extending to +280 completely abolished it (Fig. 1). These results narrow the location of P2 to region (+150/+280) (Fig. 1, rightmost dark gray rectangle).

In order to reconcile the enhancer-like magnitude of the activity mapping to region (−64/+19) and its promoter-like position spanning the *Mina* TSS, we hypothesized that it might encompass a second *Mina* promoter that is selectively targeted by a distal enhancer contained within fragment (−64/+354). To test this hypothesis, we generated the TSS-spanning fragment (−64/+80) and found it to exhibit low-level reporter activity similar to the SV40 promoter and to P2 (Fig. 1). Thus, the high-level reporter activity located within fragment (−64/+354) and mapping to region (−64/+19) is likely due to a second promoter (termed P1; Fig. 1, leftmost dark gray rectangle) targeted by a P1-specific distal enhancer (termed E1) mapping to region (+80/+354). The low- rather than high-level activity of fragment (+19/+354) – lacking P1 and containing both P2 and E1 – provides further evidence for the P1-specificity of E1.

In summary, two promoters were identified in a ∼2 kb *Mina* TSS-spanning genomic fragment: P1 in region (−64/+19) and P2 in region (+150/+280). Further, we inferred the existence of a P1-specific enhancer E1 located in region (+80/+354).

### Four Sp1/3 Binding Sites Spanning the Mina P1 Promoter

**Figure 2:**
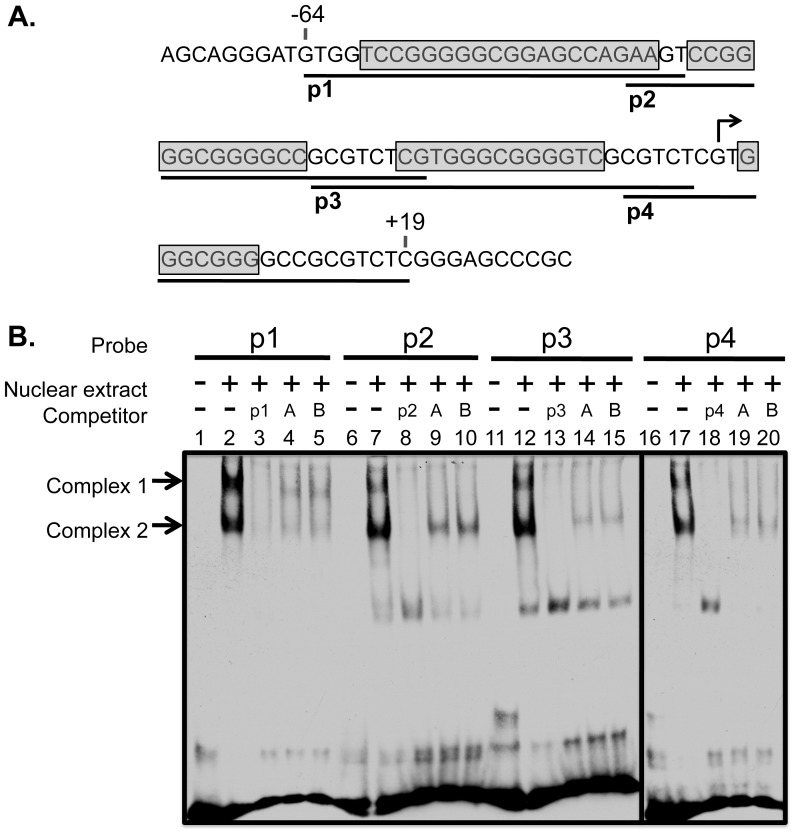
Electrophoretic mobility shift (EMSA) analysis of four consensus Sp1/3 binding sequences comprising the *Mina* P1 promoter. (A) Shown is the sequence of the *Mina* P1 promoter mapped to region (−64/+19) showing the TSS (bent arrow), the four consensus Sp1/3 binding sequences (boxed and shaded), and probes p1–p4 each containing a single Sp1/3 site (underlined and labeled). (B) Representative EMSA assay showing nucleoprotein complexes 1 and 2 (arrows) formed by combining nuclear extracts from EL4 cells with 5′ biotin labeled probes p1–p4 in the absence or presence of a 100-fold molar excess of unlabeled probes containing autologous (p1–p4) and heterologous (A and B corresponding to Sp1A and Sp1B, respectively, from the OX40 promoter [16]) Sp1/3 consensus sequences. Data are representative of 2 experiments.

Given its TSS-spanning location and its specific regulation by E1, we decided to focus on characterizing P1. Using Transfac software (Biobase), we identified a cluster of four predicted Sp1/3 binding sites spanning region (−64/+19) (Fig. 2A). To begin exploring whether any of these sites were functional, we performed gel shift assays with DNA probes (p1–p4) spanning each site and nuclear extract from the Mina-expressing EL4 murine thymoma cell line (Fig. 2A). Each of the four probes formed a similar pair of nucleoprotein complexes (Fig. 2B, complex 1 and 2 as indicated by arrows). Formation of the paired complexes was inhibited in the presence of a 100-fold molar excess of unlabeled cognate probe, as well as two known Sp1-binding sequences from the OX40 promoter [16] (Fig. 2B, Sp1A and Sp1B). These results show that each of the four predicted Sp1/3 sites in *Mina* P1 can form complexes with EL4 nuclear factors.

### Binding of Sp1/3 to the Mina P1 Promoter in EL4 Cells

**Figure 3:**
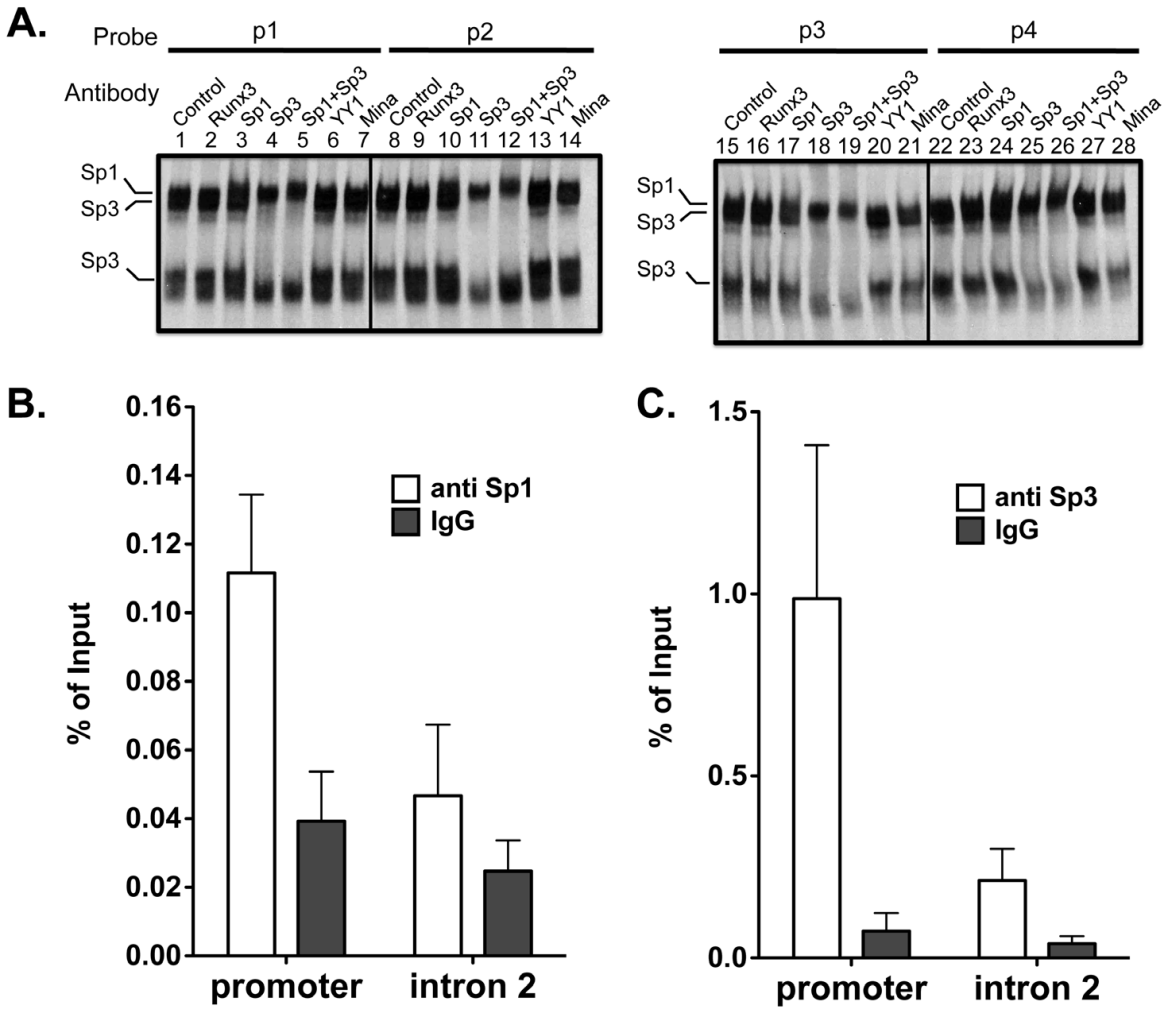
EMSA supershift analysis of Sp1/3 association with the four Sp1/3 binding sites comprising the *Mina* P1 promoter. (A) Representative EMSA assay revealing the upper and lower bands of complex 1 to contain Sp1 and Sp3, respectively, and the upper band of complex 2 to contain Sp3. Probes p1–p4 were incubated with EL4 nuclear extracts and antibodies to the indicated targets, prior to analysis by SDS-PAGE. Data are representative of 3 independent experiments. B and C) Chromatin immunoprecipitation (ChIP) analysis of Sp1 (B) and Sp3 (C) binding to the *Mina* P1 promoter region in EL4 cells. EL4 cell chromatin fragments were immunoprecipitated with antibodies against Sp1 or Sp3 (open bars) or control IgG (filled bars) and analyzed by qPCR. Promoter is the *Mina* P1-proximal promoter region; Intron 2 is located in *Mina* intron 2, ∼6 kb 3′ of the *Mina* promoter. Data are from 2 (B) or 3 (C) independent experiments.

Next, we investigated whether Sp1 and/or Sp3 occurred within nucleoprotein complexes 1 and 2. Using gel shift assays with *Mina* P1 probes and EL4 nuclear extract we found that antibodies against YY1, Runx3 and Mina failed to perturb either complex (Fig. 3A). By contrast, an Sp1-specific antibody caused a super-shift of the upper band of the complex 1 doublet, while an Sp3-specific antibody abolished the lower band of the complex 1 doublet and the upper of band of the complex 2 doublet. Combining antibodies against Sp1 and Sp3 led to a summation of their individual effects on complexes 1 and 2 (Fig. 3A). Together, these data indicate that probes p1–p4 each form 3 distinct nucleoprotein complexes, one containing Sp1 and two containing Sp3.

Next, to examine whether Sp1 and/or Sp3 bind the *Mina* promoter in EL4 cells, we performed chromatin immunoprecipitation experiments using control IgG and antibodies against Sp1 and Sp3. As shown in Figure 3B and C, a *Mina* intron 2 sequence ∼6 kb downstream of the *Mina* P1 promoter was similarly enriched in chromatin immunoprecipitated with Sp1- and Sp3-specific antibodies as with control IgG. By contrast, a *Mina* P1-proximal sequence was enriched to a significantly greater extent in chromatin immunoprecipitated with Sp1 or Sp3 antibodies as compared to control IgG. These data indicate that both Sp1 and Sp3 bind the *Mina* P1 promoter in EL4 cells.

### Regulation of Mina Expression by Sp1/3

**Figure 4:**
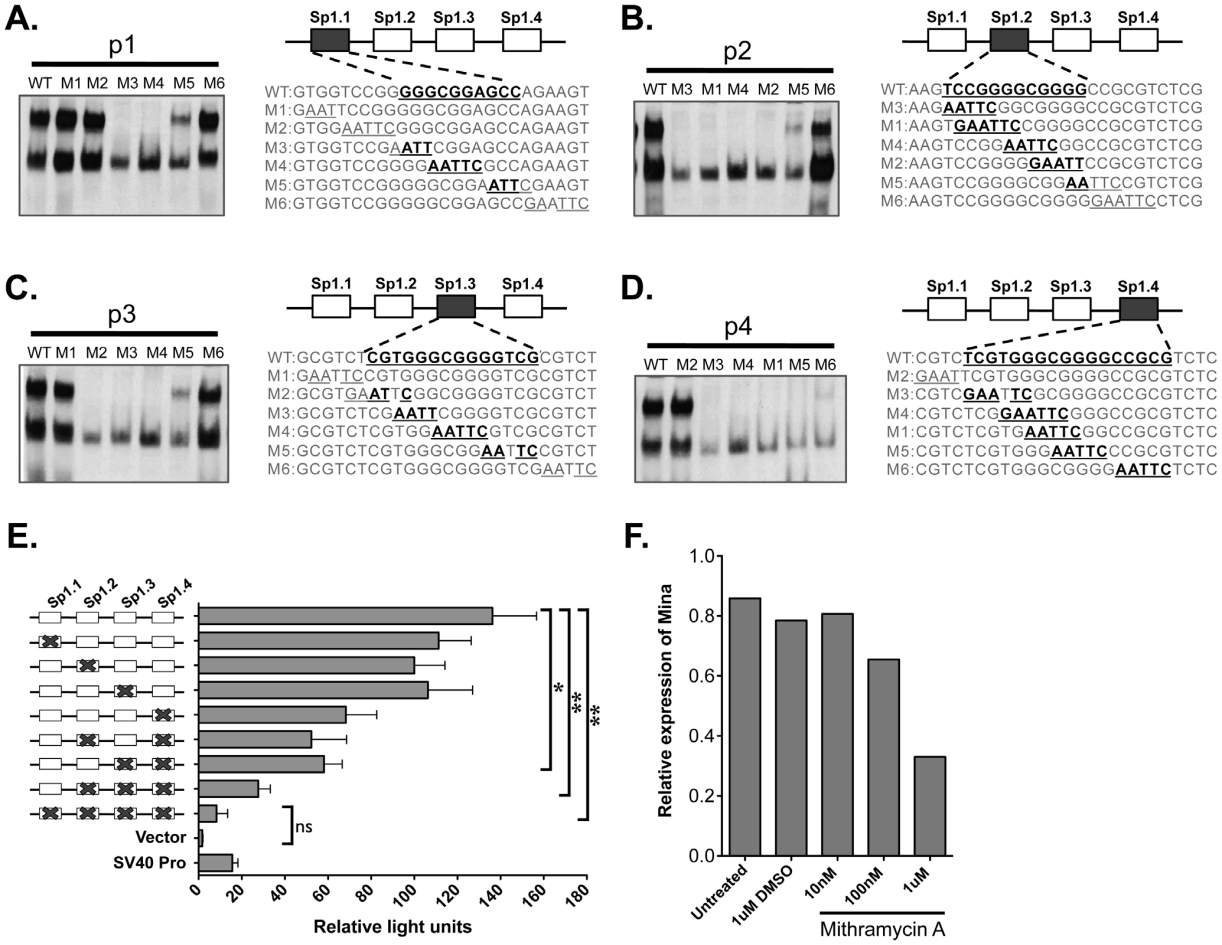
Pharmacologic and siRNA analysis of Sp1/3 activation of *Mina* promoter activity in EL4 cells. (A–D) Mapping the key nucleotides required for Sp1/3 binding to each Sp1/3 binding site (Sp1.1–Sp1.4) in the *Mina* P1 promoter. For each site, gel shift assays were performed with wild type (WT) and mutant (M1–6) probes (comprising an overlapping set of mutations, underlined), as shown to the right of each gel shift assay image. Nucleotides critical for Sp1/3 binding are bolded. Data are representative of 2 independent experiments. (E) All four Sp1/3 binding sites act additively to activate *Mina* promoter activity. Cloned into a firefly luciferase reporter construct, the four Sp1/3 sites of a WT *Mina* P1 promoter (spanning position −64 to +151) were mutated individually or in combinations, as indicated (Xs) in the schematic to the left of the bar graph depicting reporter activity. Data are presented as the mean and SEM from 3 independent experiments. Statistical significance was determined by the student’s t-test (ns, not significant; *p<0.05; **p<0.01). Vector is the PGL3 basic vector; SV40 Pro is the PGL3 basic vector containing the SV40 promoter. (F) Effect of Sp1 inhibitor Mithramycin A on *Mina* transcript level. EL4 cells were treated for 24 hr with 0, 10 nM, 100 nM, 1 uM Mithramycin A or 1 µM DMSO carrier prior to RNA harvest and analysis of *Mina* mRNA level by quantitative RT-PCR.

To extend our cell-free and cellular evidence that Sp1 and Sp3 bind to the Sp1/3 sites in the *Mina* P1 promoter, we asked next whether Sp1/3 factors regulate *Mina* P1 promoter activity. First, to map the key nucleotides required for Sp1/3 binding to each of the four Sp1/3 sites (Sp1.1–1.4), we performed gel shift assays on sets of six probe-spanning mutants. For Sp1.1, we found that probe p1 mutants M3, M4 and M5 exhibited impaired formation of complexes 1 and 2 (Fig. 4A). Similarly, we identified mutants of p1, p2 and p3 that disrupted Sp1/3-binding to Sp1.2, Sp1.3 and Sp1.4, respectively (Fig. 4B–D). Together, these data provide further evidence that each Sp1/3 site is functional for Sp1/3 binding and set the stage for functional analysis.

To explore whether Sp1/3 regulates *Mina* P1 promoter activity via any of its four Sp1/3 sites, we performed site-directed mutagenesis to introduce the relevant mutations (as determined by gel shift assay above) in each or combinations of the four Sp1/3 sites (Fig. 4E). Individual Sp1/3 site mutations did not significantly decrease reporter activity. By contrast, combinations of two or three Sp1/3 site mutations led to impaired reporter activity, while simultaneous mutation of all four Sp1/3 sites completely abolished P1 promoter activity (Fig. 4E). Together, these data show that binding of Sp1/3 to each of the four Sp1/3 sites contributes additively to the activation of the *Mina* P1 promoter.

**Figure 5:**
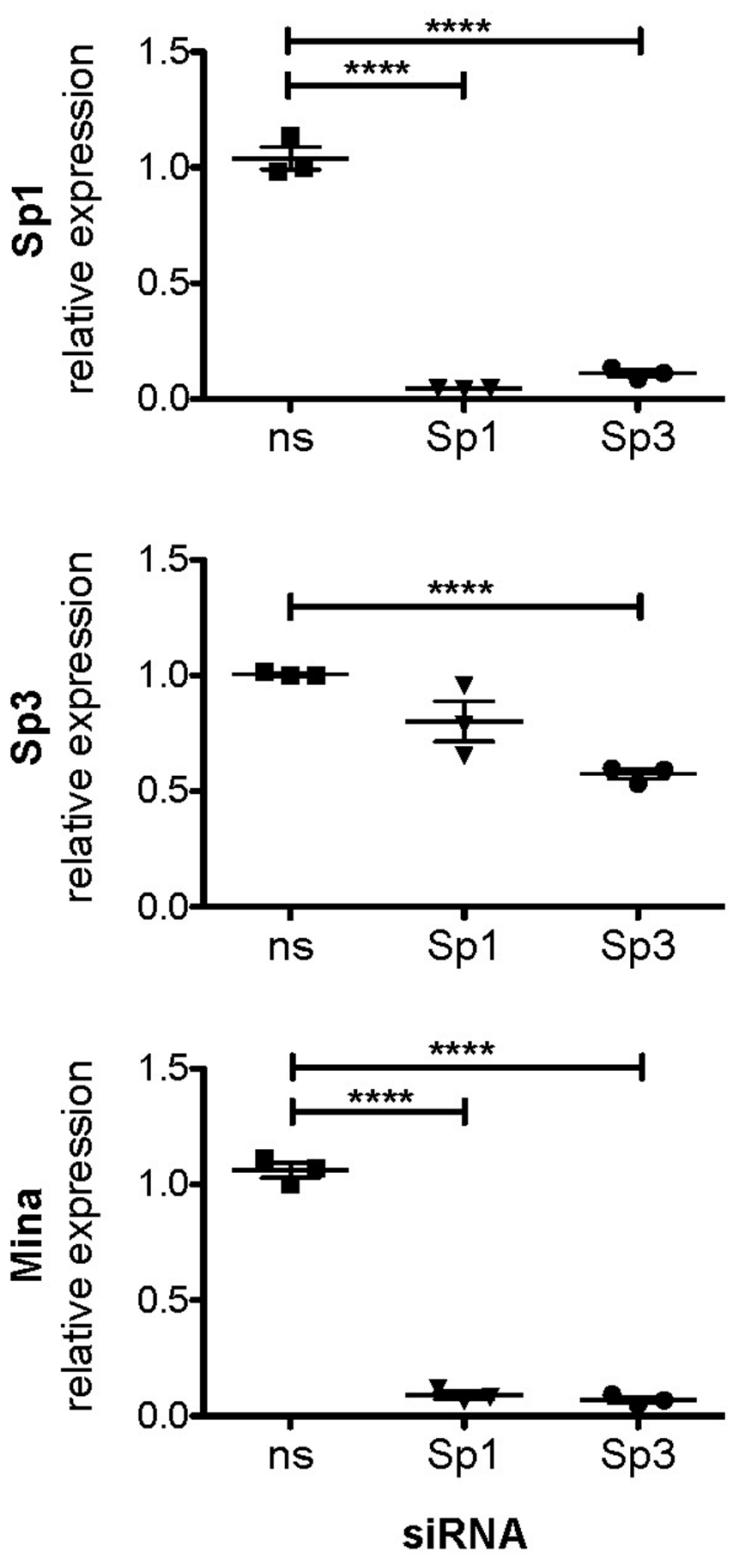
Effect on Mina expression of siRNA knockdown of Sp1/3. EL4 cells were transfected with GAPDH- and two different Sp1/3-specific as well as one non-specific Accell SMART pool siRNA and cultured for 72 hours, prior to real time RT-PCR analysis of relative mRNA levels of GAPDH, Sp1, Sp3 and Mina. Shown is a representative result from 3 independent experiments. Statistical significance of triplicate measurements was determined by the student’s t-test (****p<0.0001).

To explore whether Sp1/3 contributes to endogenous *Mina* transcription in EL4 cells, we treated cells for 24 h with Mithramycin A or vehicle alone. Mithramycin A is known to specifically inhibit Sp1 binding to its consensus GC-rich binding sites and impair its ability to promote transcription [17]}. In cells treated with 1 µΜ Mithramycin *Mina* transcript level decreased more than 50% in comparison to control-treated cells (Fig. 4F). Mithramycin did not inhibit *Hprt* expression (data not shown). To further extend these results, we performed siRNA knockdown of Sp1/3 in EL4 cells. Treatment of cells with non-specific siRNA had no detectable effects on *Sp1*, *Sp3* or *Mina* expression (Fig. 5). An Sp1-targeted siRNA abolished *Sp1* but not *Sp3* expression whereas an Sp3-targeted siRNA was found to impair expression of both *Sp3* and *Sp1*. Strikingly, both the Sp1- and Sp3-targeted siRNAs abolished *Mina* expression. Together, these data suggest that in T cells *Mina* gene transcription from its P1 promoter requires Sp1.

### Binding of Sp1/3 to the H3K4me3-enriched Mina P1 Promoter in Primary T Cells

**Figure 6:**
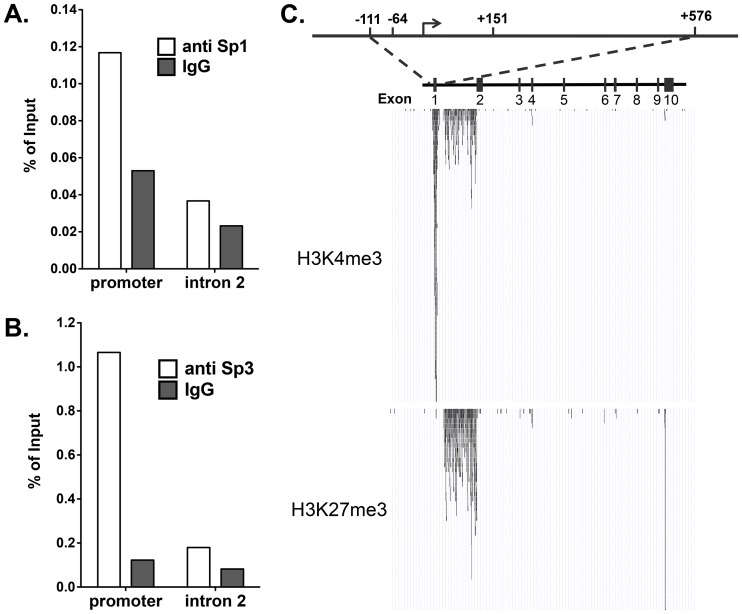
Sp1 and Sp3 binding to the *Mina* promoter in resting CD4+ T cells. (A) The *Mina* promoter was marked as an epigenetically active region in resting CD4+ T cells. Chromatin fragments from CD4^+^CD62L^Hi^CD44^−^CD25^−^ T cells of C57BL/6 mice were immunoprecipitated with antibody to either H3K27me3 or H3K4me3, and then subjected to high-throughput sequencing (ChIPseq). The ChIPseq signals from both anti-H3K27me3 (upper panel) and anti-H3K4me3 (lower panel) are shown with correlations to the *Mina* locus. The depth of the peaks in the histograms corresponds to the number of reads mapped to the location. A schematic of the *Mina* locus is shown above the histograms. Each box represents an exon. The region that enriched for H3K4me3 but was devoid of H3K27me3 modifications is enlarged and shown above the *Mina* locus schematic. (B–C) ChIP assay confirmed the binding of Sp1 and Sp3 to the *Mina* promoter in EL4 cells. Chromatin fragments from CD4^+^CD62L^Hi^CD44^−^CD25^−^ T cells of BALB/c mice were immunoprecipitated with antibodies against Sp1 (B, open bars), Sp3 (C, open bars), or their corresponding IgG controls (B–C, filled bars). Promoter: *Mina* promoter region; Intron 2: an Intron 2 region located ∼6 kb downstream of the *Mina* promoter.

We chose to explore Sp1/3 binding to the endogenous *Mina* promoter in primary naïve (CD4^+^CD25^−^CD44^low^CD62L^high^) CD4 T lymphocytes as they are known to express *Mina* at high level [1]. We assessed the enrichment of *Mina* P1 promoter-proximal and distal intron 2 sequences in chromatin immunoprecipitated with control IgG and antibodies against Sp1 and Sp3. As was the case with EL4 cells (Fig. 3B and C) we found that in primary naïve CD4 T cells both Sp1 and Sp3 bound to the *Mina* P1 promoter but not to distal intron2 (Fig. 6A and B).

Histone modifications are implicated in regulating gene expression by modulating chromatin structure, DNA accessibility and recruitment of transcription regulatory machinery [18], [19]. Tri-methylation of histone 3 at lysine 4 (H3K4me3) marks the promoters of actively transcribed genes, whereas tri-methylation of histone 3 at lysine 27 (H3K27me3) is associated with gene repression and silenced chromatin [20]. To determine whether the epigenetic landscape of the *Mina* locus correlated with its transcriptional status and Sp1/3 binding to the P1 promoter, chromatin fragments from naive CD4 T cells of C57BL/6 mice were immunoprecipitated with H3K4me3 or H3K27me3 antibody and subjected to high-throughput sequencing (ChIPseq). In region (−111/+576), spanning the location of both the P1 and P2 *Mina* promoters, we observed a striking peak of H3K4me3 enrichment (Fig. 6C), consistent with the chromatin structure in this region being open and permissive for Sp1/3 binding. Interestingly, although H3K27me3 and H3K4me3 are usually enriched at inactive and active chromatin regions, respectively, in *Mina* intron 1 both marks were co-enriched (Fig. 6C). Such so-called bivalent domains have been suggested to poise genes for either activation or repression during lineage commitment in hematopoietic stem cells [21].

## Discussion

In the current study we sought to characterize the *Mina* promoter region by exploring the transcriptional regulatory elements occurring within a 2 kb interval spanning the *Mina* TSS. We identified two promoters, termed P1 and P2, respectively mapping to region (−64/+19) spanning the TSS and to region (+150/+280) spanning exon 1 and intron 1. Three main murine *Mina* mRNA isoforms are documented in AceView [22]. The first of these initiates 14 bp downstream of P2. The 2^nd^ and 3^rd^ initiate, respectively, 11 bp downstream of and within P1. The correlation between the locations of P1 and P2 mapped in our study and the TSSs documented in AceView support our finding that *Mina* contains 2 promoters. Interestingly, we detected an enhancer-like element (termed E1) occurring within region (+80/+150) that could strongly promote reporter activity from P1 but not P2. P1 was found to contain four functional Sp1/3 sites that acted synergistically to promote reporter activity and bound both Sp1 and Sp3 in EL4 cells and primary CD4 T cells. Pharmacological inhibition of Sp1/3 binding in EL4 cells diminished the level of *Mina* transcription. Finally, the epigenetic landscape of H3K4me3 and H3K27me3 modifications (marking transcriptionally active and silent chromatin regions, respectively) across the *Mina* locus in primary CD4 T cells revealed a striking peak of H3K4me3 at the promoter region and an unexpected bivalent domain (simultaneously enriched in both H3 modifications) spanning most of intron 1. Taken together, our study provides strong support for a central role of Sp1/3 factors in regulating *Mina* transcription through binding to a cluster of 4 Sp1/3 sites in the *Mina* P1 promoter.

The precise location of the E1 enhancer (up- or down-stream of P2), the mechanism underlying its specificity for P1 versus P2, the nature of the transcription factor(s) that bind to and mediate P2 promoter activity and the relative roles of Mina P1 and P2 are currently under investigation. Interestingly, we detected a potential myeloid zinc finger 1 (Mzf1) binding site in P2 (data not shown). Mzf1 is preferentially expressed in myeloid cells and controls hematopoiesis [23]. It is tempting to postulate that through P2 Mzf1 may control *Mina* transcription in dendritic cells, another cell type where Mina is highly expressed and may function.

Sp1 and Sp3 are well-known transcription factors that modulate transcription of TATA-less genes by interacting directly with and mediating recruitment to basal transcription machinery [24]–[27]. They are deregulated in various types of human cancer. Sp1 mRNA and DNA-binding activities are shown to increase in epithelial tumors, suggesting that increased Sp1 activity contributes to skin tumor progression [28]. Further, Sp1/3 is constitutively overexpressed in pancreatic and gastric cancers and many signal transduction pathways terminating on Sp1-regulated genes are linked to cancer progression [29]–[31]. Given that *Mina* is aberrantly expressed in many cancers, it is tempting to speculate that its transcriptional regulation by Sp1/3 may contribute to its roles in cell proliferation and oncogenesis.

Sp1/3 factors also play a prominent role in the immune system, being known to regulate transcription of cytokines and regulators of T cell homeostasis, activation and differentiation, including GM-CSF [32], IL-10 [33], [34] and OX40 [16]. Interestingly, Sp1/3 contributes to the differential expression of Fas ligand (a critical component of peripheral T cell homeostasis and cytotoxic effector mechanisms), Eta-1 (a cytokine that regulates IL-10 expression and Th1 polarization), and FUT7 (the rate-limiting enzyme for synthesis of a lymphocyte homing ligand) in Th1 and Th2 cells either through differential binding to a promoter polymorphism [35] or through differential promoter recruitment of transcription factors [36], [37]. Further, Sp1/3 regulates the promoter activity of the IL4 receptor alpha chain [38], through which IL4 exerts its critical biological effects, including promotion of Th2 differentiation. We postulate that orchestration of effector T cell differentiation by master transcriptional regulators involves Sp1/3-dependent transcription of *Mina*.

The *Mina* P1 promoter appears to be able to buffer genetic variation insofar as mutations abolishing Sp1/3 binding to as many as two binding sites still permitted a level of activity not significantly different from the WT promoter with 4 functional Sp1/3 binding sites. This might also explain why 1 µM Mithramycin A was required to impair endogenous *Mina* transcription, whereas 10-fold less was sufficient to inhibit transcription of an Sp1/3 target gene (TINF2) that contained only two Sp1/3 sites [39].

This work sets the stage for a comprehensive analysis of Mina regulatory elements (enhancers and silencers) and for the discovery of regulatory SNPs that modulate Mina expression level across strains with high and low Th2-bias.

## Supporting Information

Table S1 Electromobility shift and ChIP assay primers and probes.
(DOCX)
