# Supplementary material for: Transcriptional Activation of Mina by Sp1/3 Factors
Source: PLoS One. 2013 Dec 4;8(12):e80638. doi: 10.1371/journal.pone.0080638 (PMC3851307; doi:10.1371/journal.pone.0080638)
Supplement: Table S1 — Electromobility shift and ChIP assay primers and probes. (DOCX) [file pone.0080638.s001.docx]

Table S1

| Electromobility shift and ChIP assay probes and primers | | | | | |
| --- | --- | --- | --- | --- | --- |
| Name | | | | Sequence | 5’ biotin |
| Gel shift assay | p1 | WT | For | GTGGTCCGGGGGCGGAGCCAGAAGT | Yes |
|  |  |  | Rev | ACTTCTGGCTCCGCCCCCGGACCAC | Yes |
|  |  | M1 | For | GAATTCCGGGGGCGGAGCCAGAAGT | Yes |
|  |  |  | Rev | ACTTCTGGCTCCGCCCCCGGAATTC | Yes |
|  |  | M2 | For | GTGGAATTCGGGCGGAGCCAGAAGT | Yes |
|  |  |  | Rev | ACTTCTGGCTCCGCCCGAATTCCAC | Yes |
|  |  | M3 | For | GTGGTCCGAATTCGGAGCCAGAAGT | Yes |
|  |  |  | Rev | ACTTCTGGCTCCGAATTCGGACCAC | Yes |
|  |  | M4 | For | GTGGTCCGGGGAATTCGCCAGAAGT | Yes |
|  |  |  | Rev | ACTTCTGGCGAATTCCCCGGACCAC | Yes |
|  |  | M5 | For | GTGGTCCGGGGGCGGAATTCGAAGT | Yes |
|  |  |  | Rev | ACTTCGAATTCCGCCCCCGGACCAC | Yes |
|  |  | M6 | For | GTGGTCCGGGGGCGGAGCCGAATTC | Yes |
|  |  |  | Rev | GAATTCGGCTCCGCCCCCGGACCAC | Yes |
|  | p2 | WT | For | AAGTCCGGGGCGGGGCCGCGTCTCG | Yes |
|  |  |  | Rev | CGAGACGCGGCCCCGCCCCGGACTT | Yes |
|  |  | M1 | For | AAGTGAATTCCGGGGCCGCGTCTCG | Yes |
|  |  |  | Rev | CGAGACGCGGCCCCGGAATTCACTT | Yes |
|  |  | M2 | For | AAGTCCGGGGGAATTCCGCGTCTCG | Yes |
|  |  |  | Rev | CGAGACGCGGAATTCCCCCGGACTT | Yes |
|  |  | M3 | For | AAGAATTCGGCGGGGCCGCGTCTCG | Yes |
|  |  |  | Rev | CGAGACGCGGCCCCGCCGAATTCTT | Yes |
|  |  | M4 | For | AAGTCCGGAATTCGGCCGCGTCTCG | Yes |
|  |  |  | Rev | CGAGACGCGGCCGAATTCCGGACTT | Yes |
|  |  | M5 | For | AAGTCCGGGGCGGAATTCCGTCTCG | Yes |
|  |  |  | Rev | CGAGACGGAATTCCGCCCCGGACTT | Yes |
|  |  | M6 | For | AAGTCCGGGGCGGGGGAATTCCTCG | Yes |
|  |  |  | Rev | CGAGGAATTCCCCCGCCCCGGACTT | Yes |
|  | p3 | WT | For | GCGTCTCGTGGGCGGGGTCGCGTCT | Yes |
|  |  |  | Rev | AGACGCGACCCCGCCCACGAGACGC | Yes |
|  |  | M1 | For | GAATTCCGTGGGCGGGGTCGCGTCT | Yes |
|  |  |  | Rev | AGACGCGACCCCGCCCACGGAATTC | Yes |
|  |  | M2 | For | GCGTGAATTCGGCGGGGTCGCGTCT | Yes |
|  |  |  | Rev | AGACGCGACCCCGCCGAATTCACGC | Yes |
|  |  | M3 | For | GCGTCTCGAATTCGGGGTCGCGTCT | Yes |
|  |  |  | Rev | AGACGCGACCCCGAATTCGAGACGC | Yes |
|  |  | M4 | For | GCGTCTCGTGGAATTCGTCGCGTCT | Yes |
|  |  |  | Rev | AGACGCGACGAATTCCACGAGACGC | Yes |
|  |  | M5 | For | GCGTCTCGTGGGCGGAATTCCGTCT | Yes |
|  |  |  | Rev | AGACGGAATTCCGCCCACGAGACGC | Yes |
|  |  | M6 | For | GCGTCTCGTGGGCGGGGTCGAATTC | Yes |
|  |  |  | Rev | GAATTCGACCCCGCCCACGAGACGC | Yes |
|  | p4 | WT | For | CGTCTCGTGGGCGGGGCCGCGTCTC | Yes |
|  |  |  | Rev | GAGACGCGGCCCCGCCCACGAGACG | Yes |
|  |  | M1 | For | CGTCTCGTGAATTCGGCCGCGTCTC | Yes |
|  |  |  | Rev | GAGACGCGGCCGAATTCACGAGACG | Yes |
|  |  | M2 | For | GAATTCGTGGGCGGGGCCGCGTCTC | Yes |
|  |  |  | Rev | GAGACGCGGCCCCGCCCACGAATTC | Yes |
|  |  | M3 | For | CGTCGAATTCGCGGGGCCGCGTCTC | Yes |
|  |  |  | Rev | GAGACGCGGCCCCGCGAATTCGACG | Yes |
|  |  | M4 | For | CGTCTCGGAATTCGGGCCGCGTCTC | Yes |
|  |  |  | Rev | GAGACGCGGCCCGAATTCCGAGACG | Yes |
|  |  | M5 | For | CGTCTCGTGGGAATTCCCGCGTCTC | Yes |
|  |  |  | Rev | GAGACGCGGGAATTCCCACGAGACG | Yes |
|  |  | M6 | For | CGTCTCGTGGGCGGGGAATTCTCTC | Yes |
|  |  |  | Rev | GAGAGAATTCCCCGCCCACGAGACG | Yes |
|  | Sp1A | | For | TCTTGCCCCACCTCCATAGTTCTTATAGCC | No |
|  |  |  | Rev | GGCTATAAGAACTATGGAGGTGGGGCAAGA | No |
|  | Sp1B | | For | TTATAGCCACACCCTGCAAGGAAAA | No |
|  |  |  | Rev | TTTTCCTTGCAGGGTGTGGCTATAA | No |
| ChIP | Mina P1 promoter | | For | CCGATGGAGTACAAGCACTCTCTCAA | No |
|  |  |  | Rev | GCTCTGGCCTATGAACCTAAAGGT | No |
|  | Mina intron 2 | | For | TGCAGAGTCCTCTCCAATTCCACA | No |
|  |  |  | Rev | AAGCAGCACAAACAAGGGATGGAC | No |
